# The mechanism for the different effects of texture on yield strength and hardness of Mg alloys

**DOI:** 10.1038/s41598-017-08966-z

**Published:** 2017-08-17

**Authors:** Feilong Guo, Huihui Yu, Chenyu Wu, Yunchang Xin, Cong He, Qing Liu

**Affiliations:** College of Materials Science and Engineering, Chongqing University, Chongqing, 400030 China

## Abstract

A study regarding the effect of texture on tensile yield strength and hardness of an extruded Mg-15Gd-0.5Zr rod was performed, with a great emphasis laid on the relevant mechanisms. A 7% pre-tension along the extrusion direction (ED) in the solid solution condition was used to transform the texture from a broad distribution of basal poles with a peak approximately 45° with respect to the ED into a texture with basal poles largely perpendicular to the ED. This texture variation enhances the yield strength of peak aged sample by approximately 103 MPa, while hardly increases the peak hardness. The analysis about the ratio of the critical resolved shear stress (CRSS) to Schmid factor shows that this texture variation results in a larger fraction of grains favoring prismatic slip with a higher activation stress under tension along the ED. In contrast, the complex stress state during hardness test initiates multiple deformation modes, which renders the value of hardness insensitive to the texture variation. This different dependence of deformation modes on texture mainly accounts for the different increments in hardness and tensile yield strength.

## Introduction

Mg alloys are desirable candidates as weight-saving structural materials in automobile and aerospace industries^1^. However, Mg with a *hcp* structure has only two easy deformation modes at room temperature, basal slip and $\{10\overline{1}2\}$ twinning^2, 3^, leading to a poor cold working ability. In addition, Mg alloys are usually less strong, compared with their counterparts such as Al alloys^1, 4^. For example, the yield strengths of commercial high-strength Mg alloys (e.g. AZ80 or ZK60) mainly fall in the range of 250–350 MPa (specific strength 139–194 MPa/(g/cm^3^))^4^, while those of some commercial high strength Al alloys (e.g. 7050 or 2095) are up to 500–700 MPa (specific strength 185–259 MPa/(g/cm^3^))^1^.

Addition of rare earth (RE) can effectively enhance the yield strength of Mg alloys. For example, an extruded Mg-10Gd-5.7Y-1.6Zn-0.5Zr in the peak-aged condition exhibits a tensile yield strength of 473 MPa and an ultimate tensile strength of 542 MPa^5^. The tensile yield strength of a Mg-7Y-2.5Zn alloy prepared by rapidly solidified powder metallurgy processing even exceeds 600 MPa^6^. The high density of plate-shaped precipitates with a habit plane parallel to prismatic planes (e.g. β_1_ phase in WE54 and β phase in Mg-Y alloy), is considered to be the primary cause for the high strength in those alloys^4, 7^. Theoretical calculation by the Orowan equation shows that the CRSS increase for basal slip produced by prismatic plates is invariably larger than those produced by equivalent volume fraction of basal plates typically formed in AZ alloys^4, 7^. Addition of RE can also develop a weak recrystallized texture, forming the so-called “RE texture”. A notable example is the Mg-1Zn-1Gd hot rolled plate with basal poles tilting away from the normal direction by about 30–40°. This plate exhibits a maximum elongation up to 36%^8^.

Formation of a strong texture is an effective way to improve strength. However, the work from Li *et al*.^9^, Yang *et al*.^10^ and Robson *et al*.^11^ shows that many high strength Mg-RE alloys have a weak texture. If the weak texture could be transformed into a strong one, further enhancing the strength of those alloys would be obtained. In this work, a 7% pre-tension of a Mg-15Gd-0.5Zr extruded rod in the solid solution condition transformed the texture from soft orientations for basal slip into hard ones. This texture variation enhanced the tensile yield strength along the extrusion direction (ED) in peak aged sample by approximately 103 MPa, while hardly increased the peak hardness. A great emphasis in this paper was placed on the mechanism for the different roles of texture in tensile yield strength and hardness.

## Results

### Mechanical behavior

**Figure 1:**
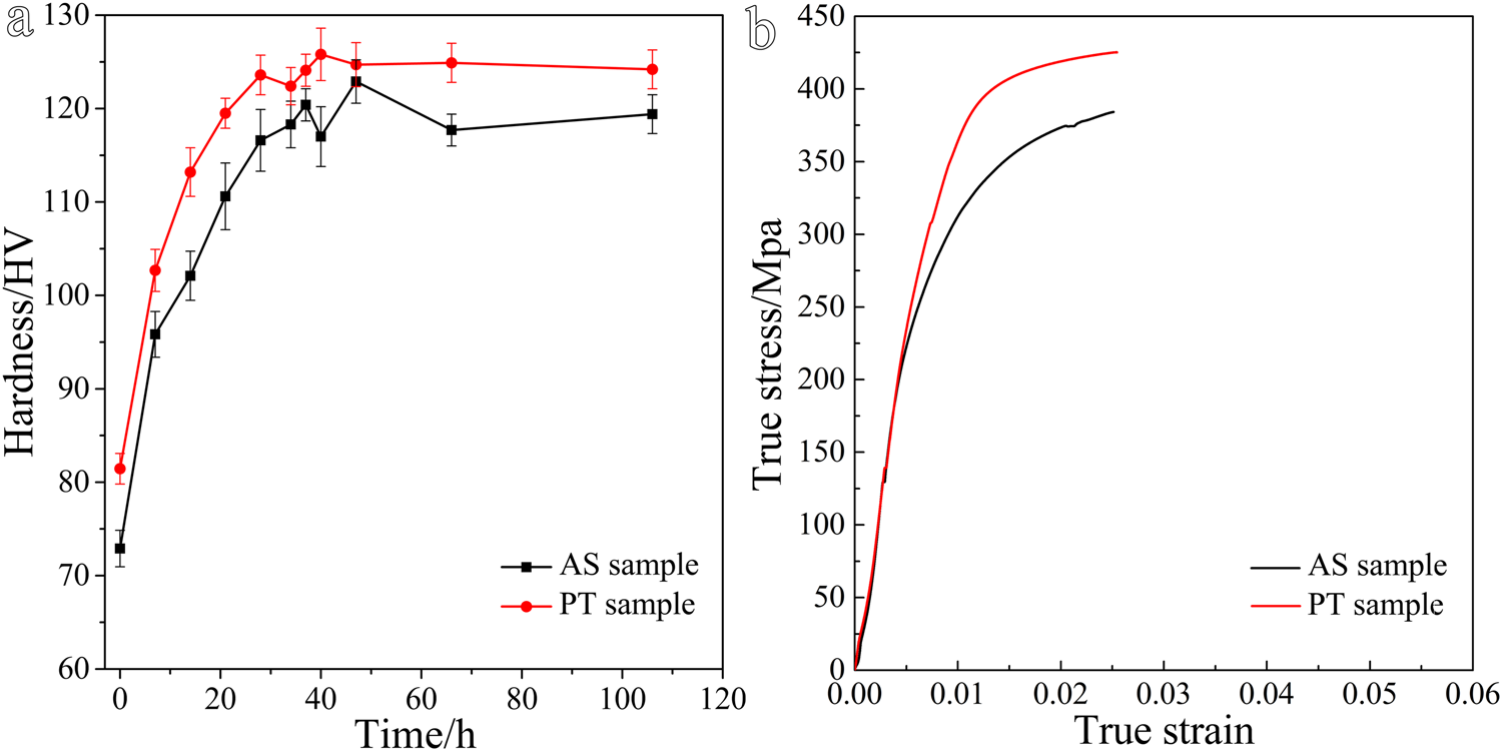
(**a**) Vickers hardness as function of time of Mg-15 Gd–0.5 Zr rods aged at 180 °C and (**b**) true stress-strain curves of peak aged sample under tension along the ED at room temperature.

Age hardening responses and tensile stress-strain curves are shown in Fig. 1, with the mechanical properties listed in Table 1. The peak hardness in pre-tensioned sample (PT sample) is about 126 Hv, quite similar to that in the initial extruded rod (AS sample), 123 Hv. In contrast, the yield strength of peak aged AS sample, 283 MPa, is much lower than that of PT sample, 386 MPa.

**Table 1:** Yield strengths, ultimate strengths and elongations of the peak aged samples under tension along the ED.

Sample	AS sample	PT sample
Yield Strength (MPa)	283 (±6)	394 (±10)
Ultimate strength (MPa)	386 (±8)	435 (±13)
Elongation to failure (%)	2.5 (±1.2)	2.3 (±0.8)

The numbers shown in parentheses are standard deviations obtained from repeated tests.

### Microstructure examined by EBSD

**Figure 2:**
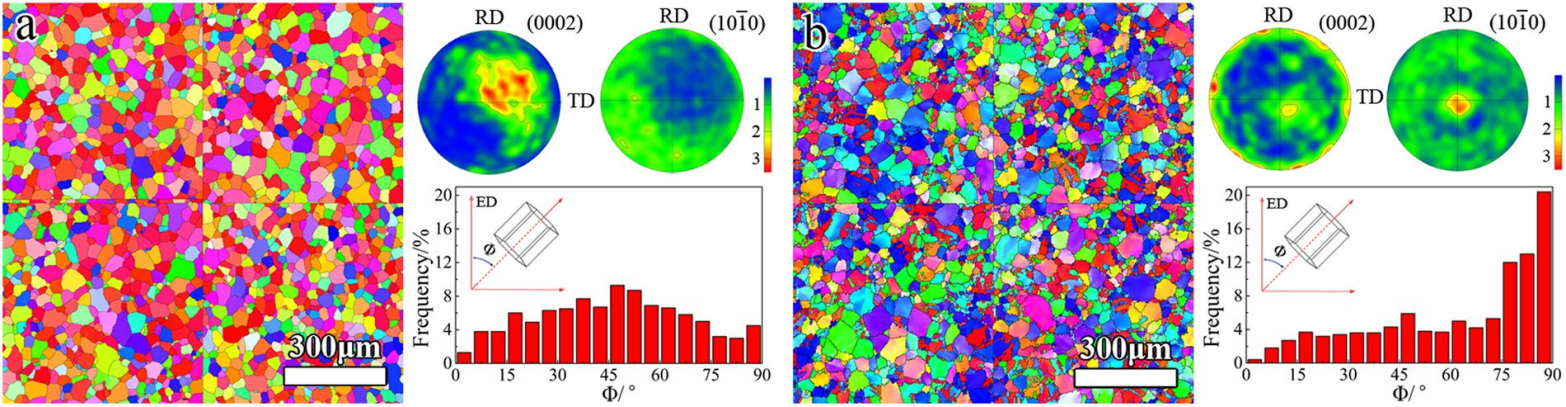
Inverse pole figure maps, pole figures and distributions of tilting angles of basal poles (Φ) with respect to the ED: (**a**) AS sample; (**b**) PT sample. Note that the RD and TD denote the radial direction and tangential direction, respectively.

Figure 2 shows the microstructure and pole figures of AS sample and PT sample. Some twins identified as $\{10\overline{1}2\}$ twins exist in PT sample (Fig. 2b). The average grain sizes are measured as about 45 μm in AS sample and approximately 42 μm in PT sample. The linear intercept method was used to measure the grain size. In this method, the linear intercept size calculated as the ratio of the length of a line to the number of intercepted grains is taken as the equivalent grain size. For the measurement of grains containing twins, the lamella spacing is considered as the equivalent grain size^12^. As seen in the pole figures, the as-used pre-tension largely varies the crystallographic orientation distribution. A quantified measurement of the angle (Φ) between the c-axis of grains and the ED is given in Fig. 2a and b. To obtain statistical results, four separate areas were indexed and analyzed for each sample. In AS sample, the Φ shows a broad distribution with a peak around 45°, while in PT sample the Φ of about 56% grains are over 70°.

### Microstructure examined by TEM

**Figure 3:**
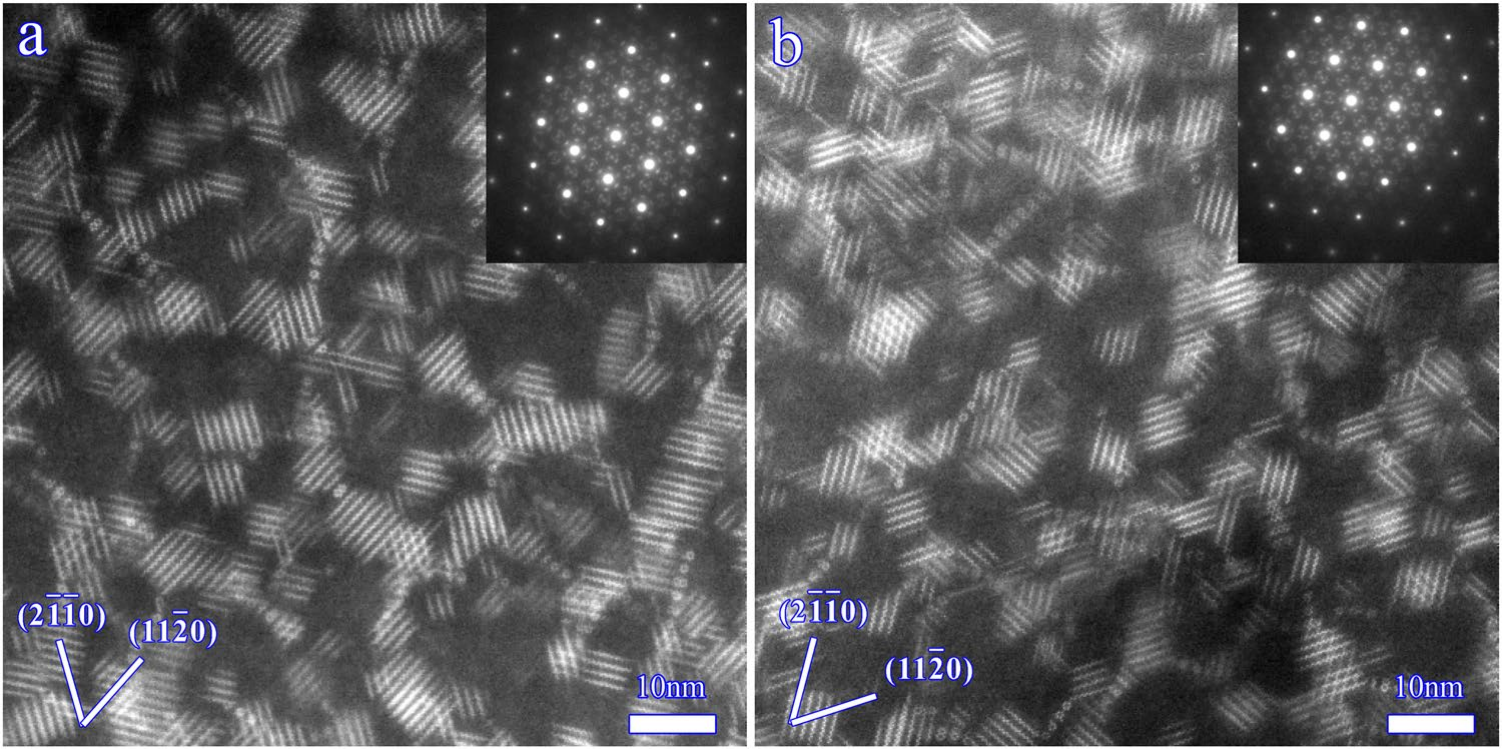
HAADF-STEM images showing the distribution and morphology of precipitates in peak aged condition in (**a**) AS sample and (**b**) PT sample. Electron beam is parallel to [0001]_α_.

High-angle annular dark-field scanning transmission electron microscopy (HAADF-STEM) images of precipitates in AS sample and PT sample in the peak aged condition are shown in Fig. 3. A large number of β′ phase exist in the two samples. The precipitate densities in PT sample and AS sample are measured as approximately 1.12 × 10^22^ m^−3^ and 1.09 × 10^22^ m^−3^, respectively. The measurement of precipitate density considers the TEM foil thickness measured by the convergent-beam electron diffraction technique^13^. The measured value is a density per unit volume. Obviously, a similar density of precipitates exists in the two samples. In fact, the 7% pre-tension is adopted in order to largely alter the texture, meanwhile does not obviously vary the density of precipitates.

## Discussion

**Figure 4:**
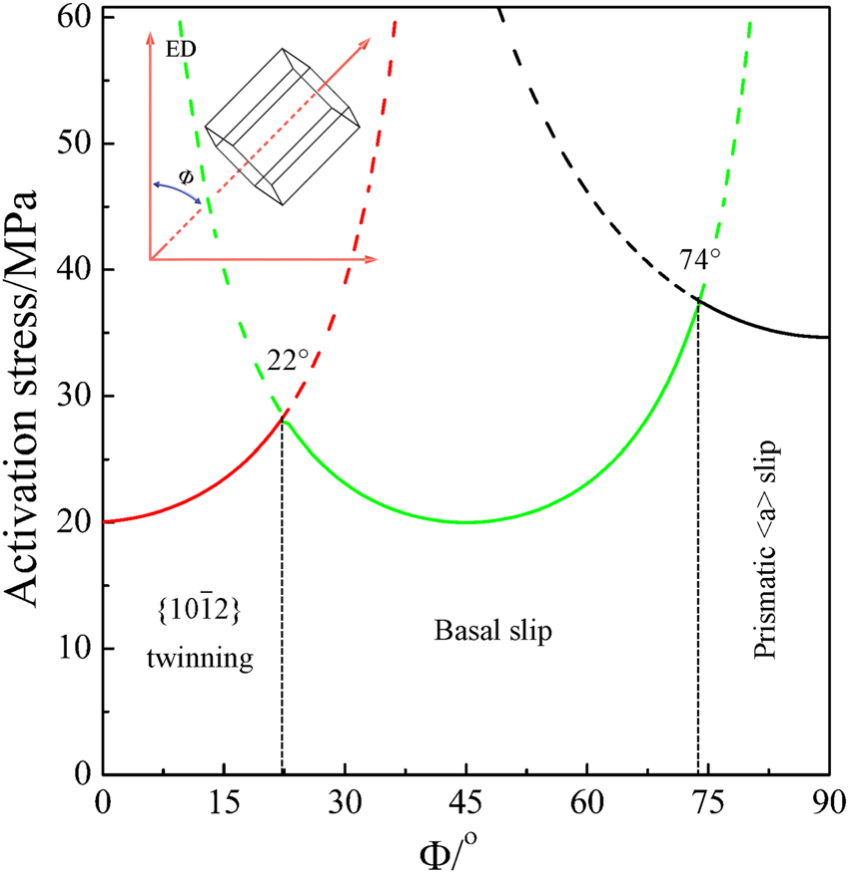
Activation stresses for the dominant deformation modes under tension along the ED as a function of the tilting angle of basal poles (Φ) with respect to the ED.

Since there are different textures between AS and PT samples as shown in Fig. 2, this would result in a variation of deformation modes under tension along the ED. The dominant deformation mode in Mg alloy is highly dependent on the angle between basal pole of a grain and the loading direction. To determine the predominant mode as a function of Φ under ED tension, the ratios of the critical resolved shear stress (CRSS) to Schmid factor (SF) for basal slip, $\{10\overline{1}2\}$ twinning and prismatic slip were calculated. Only one fixed orientation at a given Φ was calculated. The mode that gives the minimum ratio value is the dominant mode. The choice of the CRSS ratio between deformation modes is of great importance. As could be seen in most simulations, basal slip and $\{10\overline{1}2\}$ twinning are the two easiest deformation modes and possess a similar CRSS^14^. Although different CRSS ratios of basal slip to prismatic slip were used in different simulations, values of 2–3 were widely utilized for the simulation of a Mg AZ31^15–17^. Considering that addition of Gd might promote the activity of prismatic slip^18^, the CRSS ratio of 1:1:1.5 for basal slip (10 MPa): $\{10\overline{1}2\}$ twinning (10 MPa): prismatic slip (15 MPa) was used in this study. As seen in Fig. 4, the dominant mode is $\{10\overline{1}2\}$ twinning with Φ below 22°, basal slip with Φ between 22° and 74° and prismatic slip with Φ over 74°. In addition, the activation stress for prismatic slip is generally higher than that for basal slip and $\{10\overline{1}2\}$ twinning. Obviously, the dominant deformation mode and its activation stress are highly dependent on Φ. The fraction of grains favorable for basal slip, prismatic slip, and $\{10\overline{1}2\}$ twinning during tension along the ED within the two samples was calculated using the same CRSS ratio, and the results are listed in Table 2. For each sample, approximately 900 grains were used for this calculation. The fraction favoring basal slip is up to 69% in AS sample, while only about 41% in PT sample. In stark contrast, the fraction favoring prismatic slip in AS sample (15%) is greatly lower than that in PT sample (49%). As the activation stress for prismatic slip is higher than that for basal slip, the inclination of basal poses toward the direction perpendicular to the ED will enhance the yield strength during tension along the ED.

**Table 2:** The fractions of grains favoring basal slip, prismatic slip, and $\{10\overline{1}2\}$ twinning during tension along the ED.

Sample	Loading direction	Basal slip	Prismatic 〈a〉 slip	$\{\mathrm{10}\bar{{\bf{1}}}2\}$ twinning
AS sample	Tension//ED	69%	15%	16%
PT sample	Tension//ED	41%	49%	10%

The used CRSS ratio of basal slip: $\{10\overline{1}2\}$ twinning: prismatic slip is 1:1:1.5.

**Figure 5:**
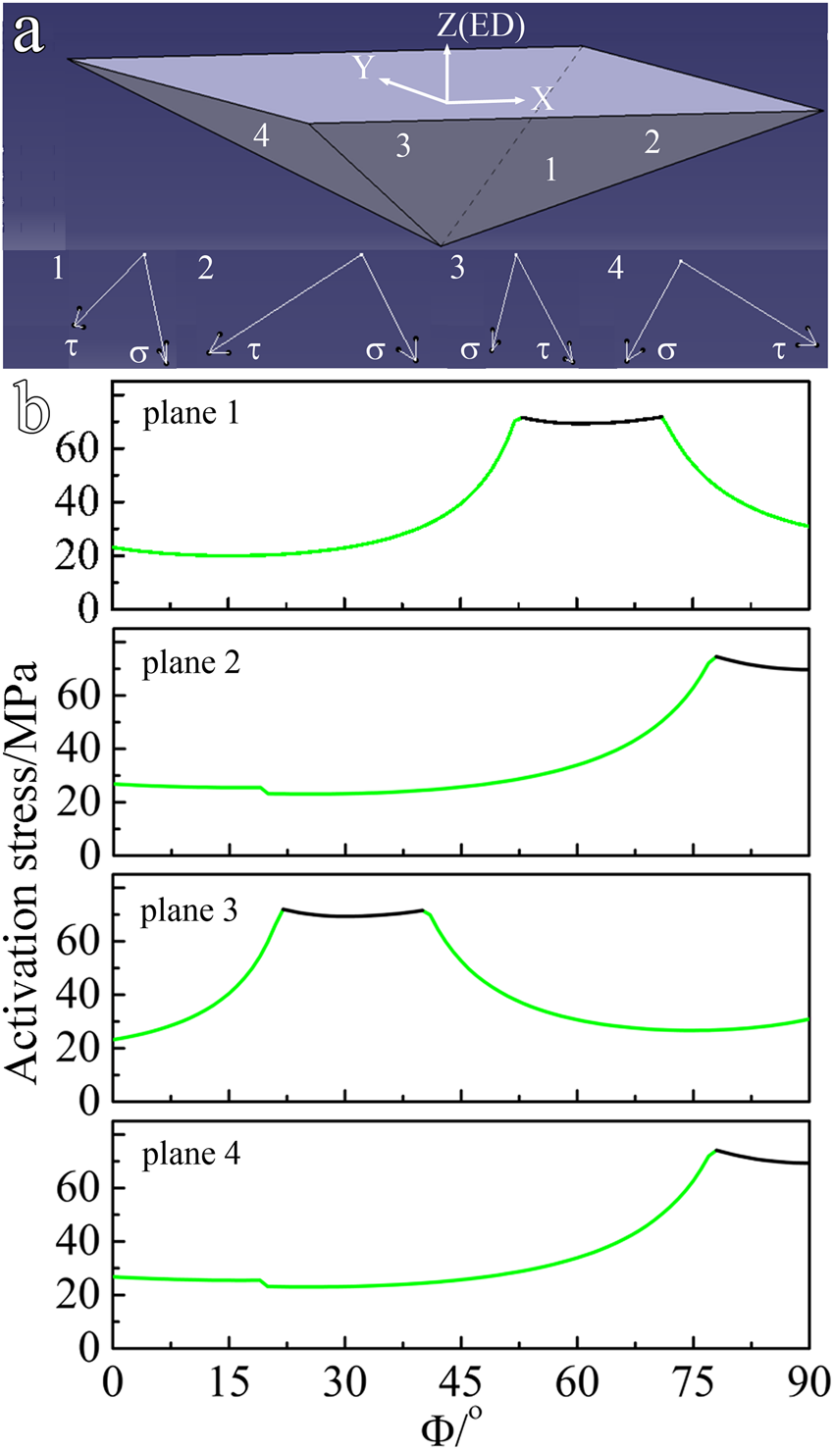
(**a**) Schematic diagram of the Vickers indenter and the direction of the compressive stress (σ) and shear stress (τ) exerted onto the sample by each pyramidal plane (planes labeled as 1, 2, 3 and 4); (**b**) activation stresses for the dominant deformation modes as a function of the tilting angle of basal poles (Φ) with respect to the ED.

Different from uniaxial tension, there exists a more complex stress state in Vickers hardness test. As shown in Fig. 5a, the indenter has four pyramidal planes (labeled as 1, 2, 3, 4), each side of which will exert a compressive stress (σ, normal to the pyramidal plane surface) and a shear stress (τ, parallel to the pyramidal plane surface) onto the sample^19^. Our simulation shows that the σ/τ near the identention surface is approximately 2:1. This ratio is similar to that predicted by Prasad *et al*.^19^. To determine the dominant deformation mode as a function of Φ during hardness tests, we also calculated the CRSS/SF for basal slip, $\{10\overline{1}2\}$ twinning and prismatic slip. However, the form of Schmid law under multi-axial stress state is different from that under an uniaxial stress state^20, 21^. For example, both the tensile stress parallel to the rolling direction and compressive stress along the normal direction are incorporated in the calculation of the SF under rolling condition^21^. Following this method, the SF for hardness tests considering both the compressive stress and shear stress is calculated using the following formula:

1 $$m=\,\cos \,\alpha \,\cos \,\beta -\,\cos \,\varphi \,\cos \,\phi $$

where *α*/*ϕ* is the angle between the shear stress/compressive stress and the slip plane normal, and *β*/*φ* is the angle between the shear stress/compressive stress and the slip direction.

The diamond indenter has four pyramidal planes and each plane contacts the sample and generates the sample deformation separately. For each plane, one curve regarding the predominant modes as a function of Φ was obtained and shown in Fig. 5b. The same CRSS ratio was used. It should be highlighted that only one fixed orientation at a given Φ in Y-Z plane in Fig. 5a was calculated. Again, the activation stress for prismatic slip is higher than that for basal slip. It is apparent that there is a huge difference in the dependence of dominant modes on Φ between each indenter plane. For example, at a Φ = 30°, planes 1, 2 and 4 activate basal slip, while plane 3 initiates prismatic slip; at a Φ = 80°, basal slip is activated by planes 1 and 3, while prismatic slip by planes 2 and 4. It could be inferred that if the indention is in the interior of only one grain, both basal slip and prismatic slip might be activated; when the indention covers two or more grains, basal slip might be activated in some grains while prismatic slip in others. This renders that for a given Φ, multiple deformation modes could be activated during hardness tests, making the value of hardness insensitive to the variation of Φ.

**Figure 6:**
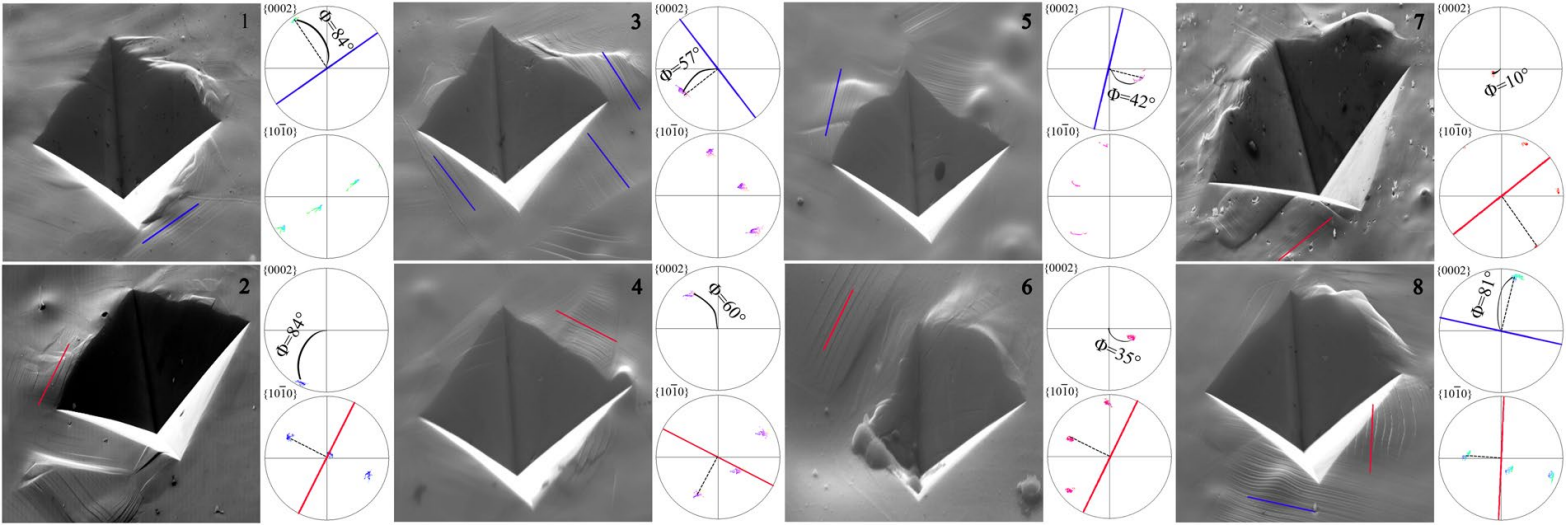
Slip trace analysis illustrating the activated slips during Vickers hardness test. The lines on the pole figures correspond to the trace of the slip plane closest to the observed slip trace (basal slip trace - blue line, prismatic slip trace - red line). Φ is the angle of basal pole away from the ED.

To further confirm this, slip trace analysis was conducted after hardness test. The activated slip system was identified based on the best match of the observed slip traces with the theoretical slip trace deduced from EBSD measurement. Whenever more than one slip could be chosen, the one with the highest SF was chosen. As seen in Fig. 6, at a similar Φ (e.g. regions 1 and 2 or regions 3 and 4 or regions 5 and 6), both prismatic slip and basal slip could be initiated. As seen in region 8, both basal and prismatic slips are activated in one grain. The results from slip trace analysis are in well accordance with those in Fig. 5b.

It is well established that both hardness and strength are sensitive to the variation of grain size, precipitate density and dislocation density^22, 23^. The observations in Figs 2 and 3 reveal that there is a similar grain size and number density of precipitates in AS and PT samples. The dislocation density might be increased to some extent by the 7% pre-tension. The aging at 180 °C for 40 h will induce a pronounced recovery of the dislocations. In addition, Clark *et al*. reported that precipitation occurs on dislocations^24^. However, part of dislocations might still remain in the PT sample, since the length scale of a dislocation line is much larger than that of precipitates. Generally, the low-angle boundary (misorientation angle of 2–4°) is associated with the dislocation density in deformed metals. Here, we measured the low-angle boundary density in PT sample as about 0.03852 μm^−1^. To measure this low-angle boundary density, a picture containing only low-angle boundaries was exported from the EBSD data and the Image-Pro Plus software was used to measure the length of low-angle boundaries. For each sample, about 900 grains were used for the measurement. The value in PT sample is much lower than that reported in a hot rolled Mg AZ31 plate with 20% reductions^25^. Therefore, the contribution of work hardening to the hardness and yield strength of peak aged sample is quite limited in the present study. That is, pre-tension affects the yield strength and hardness mainly by texture variation. As discussed previously, the texture variation allows a greater fraction of grains favoring prismatic slip with a higher activation stress in PT sample (49%) than that in AS sample (15%) during tension along the ED. In stark contrast, the value of hardness is insensitive to texture variation. At last, the texture variation after pre-tension greatly increases the yield strength, while hardly enhances the hardness. It is found that when the hardness value is increased, the yield strengths of *fcc* or *bcc* metals would be enhanced. In Mg alloys with a *hcp* structure, the relation between yield strength and hardness is complicate. Our results show that the yield strength of Mg alloys is highly dependent on texture, while the hardness is insensitive to texture. Therefore, the relation between yield strength and hardness in Mg alloys might change with texture. The high texture dependence of yield strength in Mg alloys also allows texture modification to be an effective way to improve the yield strength.

## Conclusions

In summary, a 7% pre-tension of Mg-15Gd-0.5Zr rod in solid solution condition was used to transform the texture from soft orientations favorable for basal slip into hard ones. The effect of texture on tensile yield strength and hardness was investigated. Several conclusions are reached as follows:

1. The 7% pre-tension transforms the broad distribution of basal poles with a peak approximately 45° with respect to the ED into a texture with 56% basal poles away from the ED over 70°. The pre-tension enhances the yield strength in peak aged condition by approximately 103 MPa, while hardly increases the peak hardness.
2. The different dependence of deformation modes on texture mainly accounts for the different increases in tensile yield strength and hardness. The calculation of CRSS/SF shows that, in PT sample compared with AS sample, 34% more grains favor prismatic slip with a higher activation stress under ED tension. In contrast, the complex stress state during hardness test often initiates multiple deformation modes at a certain Φ, which renders the value of hardness insensitive to the texture variation.

## Methods

### Sample preparation

An as-cast Mg alloy billet (230 mm in diameter) with a nominal composition of Mg-14Gd-0.5Zr (wt.%) was machined into cylinder with a diameter of 80 mm. This cylinder was homogenization at 505 °C for 24 h and extruded at 450 °C using an extrusion ration of 25:1 and extrusion rate of 0.5 mm s^−1^. The final extruded bar with a diameter of 16 mm was quenched in water immediately after exiting the die and subsequently subjected to a solution treatment at 500 °C for 2 h. A part of the extruded rods were pre-tensioned along the ED by about 7%. Both the as-extruded rod (the designated AS sample) and the pre-tensioned one (the designated PT sample) were then aged at 180 °C.

### Mechanical test

Vickers hardness testing using a 500 g load and a holding time of 15 s was carried out on a Shimadzu Vickers hardness testing system (HMV-G20). Tensile tests along the ED at room temperature were performed on a MTS machine at a strain rate 10^−3^ s^−1^. The specimens were dog bone shaped samples with a gauge length 40 mm and diameter 8 mm.

### Microstructure observation

Electron backscattered diffraction (EBSD) mapping using a step size of 1 μm was performed on a FEI Nova 400 SEM equipped with a HKL Channel 5 system. The precipitates were examined by transmission electron microscopy (TEM) on a FEI-F20 TEM. Thin foils for TEM observation were prepared by mechanical grinding and subsequent ion beam thinning (Gatan PIPS 691).

### Finite element simulation

Finite element simulations were conducted using the commercial available ABAQUS finite element software. In the simulation, the static friction coefficient was set to be 0.1 and the dynamic friction coefficient was varying from 0.04 to 0.06. The indenter was modeled as a near-rigid solid. At least 20 elements were in contact with the associated indenter and the Mg alloy under the maximum loading situation.
